# Potential of Anti-MUC1 Antibodies as a Targeted Therapy for Gastrointestinal Cancers

**DOI:** 10.3390/vaccines8040659

**Published:** 2020-11-05

**Authors:** Mukulika Bose, Pinku Mukherjee

**Affiliations:** Department of Biological Sciences, University of North Carolina, Charlotte, NC 28223, USA; pmukherj@uncc.edu

**Keywords:** MUC1, immunotherapy, monoclonal antibody, gastrointestinal cancers, CAR-T cells

## Abstract

Gastrointestinal cancers (GI) account for 26% of cancer incidences globally and 35% of all cancer-related deaths. The main challenge is to target cancer specific antigens. Mucins are heavily O-glycosylated proteins overexpressed in different cancers. The transmembrane glycoprotein MUC1 is the most likeable target for antibodies, owing to its specific overexpression and aberrant glycosylation in many types of cancers. For the past 30 years, MUC1 has remained a possible diagnostic marker and therapeutic target. Despite initiation of numerous clinical trials, a comprehensively effective therapy with clinical benefit is yet to be achieved. However, the interest in MUC1 as a therapeutic target remains unaltered. For all translational studies, it is important to incorporate updated relevant research findings into therapeutic strategies. In this review we present an overview of the antibodies targeting MUC1 in GI cancers, their potential role in immunotherapy (i.e., antibody-drug and radioimmunoconjugates, CAR-T cells), and other novel therapeutic strategies. We also present our perspectives on how the mechanisms of action of different anti-MUC1 antibodies can target specific hallmarks of cancer and therefore be utilized as a combination therapy for better clinical outcomes.

## 1. Global Burden of GI Cancers

Gastrointestinal (GI) cancers collectively refer to cancers of the esophagus and stomach (gastroesophageal cancers), the colon and rectum (colorectal cancers), pancreas, liver, gallbladder, small intestine, appendix, and anus. Following lung cancer (18.4%), colorectal cancer (9.2%), stomach cancer (8.2%), and liver cancer (8.2%) form the leading causes of cancer-related deaths worldwide [1].

According to the American Cancer Society (ACS) (www.cancer.org), gastrointestinal (GI) cancers have the highest incidence and are the second leading cause of cancer-related deaths in the United States. Esophageal cancer is the seventh most commonly diagnosed cancer and the 6th leading cause of cancer-related deaths worldwide [1]. It is often detected late and there are usually no early symptoms. The overall five-year survival rate for advanced esophageal cancer in the United States is about 15% [2]. Stomach cancer, or gastric cancer, is the fifth most common cancer in the world and the second highest cause of cancer-related deaths globally [3].

Pancreatic Cancer is the twelfth most common cancer globally and the seventh leading cause of cancer-related deaths [1]. However, in the US it is the third leading cause of cancer-related deaths and is projected to become the second by the end of the year 2020. Most of the pancreatic tumors are detected at a very advanced stage thus making it a lethal disease. It has a dismal 5% 5-year survival rate globally, a mean life expectancy of <6 months, and a high degree of resistance to standard therapy. In the US the five-year survival rate is 9%, which is the lowest of all major cancers. Liver cancer is the sixth most commonly diagnosed cancer and the fourth leading cause of cancer-related deaths worldwide [1]. Colorectal cancer is the third most common cancer worldwide and the second leading cause of cancer mortality [1].

Chemotherapy and radiation therapy alone or in combination with surgery remain the main modes of treatment so far. However, various immunotherapies are undergoing trials with monoclonal antibodies, combination therapies, CAR-T cell, dendritic cell therapies etc. In the last 40 years, the incidence and mortality of GI cancers have only increased without improvement in therapy. The main challenge is to target specific antigens that are not expressed in normal tissues. Mucins have always been shown to be key immunological players in various chronic and infectious diseases including cancer. In this review, we will provide a detailed overview of various immunotherapies developed against the mucin protein MUC1 in GI cancers including monoclonal antibodies, CAR-T cells and bi-specific antibodies that have successfully been through preclinical and clinical trials. We will also provide perspectives on how some of these antibodies target specific hallmarks of cancer so that they can be combined with other drugs for better outcomes in the clinic.

## 2. MUC1 as a Target Antigen in GI Cancers

### 2.1. Structure of MUC1

Mucins are high molecular weight glycoproteins and their main function is to lubricate epithelial cell surfaces and protect them against invading pathogens [4]. Mucins are broadly divided into secretory gel-forming mucins (MUC2, MUC5AC, MUC5B, MUC6, MUC7 and MUC19, as protective barriers for underlying mucosal cells) and membrane-bound mucins (MUC1, MUC3A, MUC3B, MUC4, MUC12, MUC13, MUC15, MUC16, MUC17, and MUC20) that have a transmembrane, N-terminal extracellular domain (ECD), and a C-terminal cytoplasmic tail. Secretory gel-forming mucins work as protective barriers for underlying mucosal cells, while membrane-bound mucins also play a key role in cell signaling pathways and cellular interactions [4,5,6].

Mucin 1 or MUC1 (also known as episialin, PEM, EMA, H23Ag, MCA, and CA15-3) was the first transmembrane mucin to be identified and structurally characterized [7,8,9,10]. MUC1 is a single pass type I transmembrane glycoprotein with a hyperglycosylated extracellular N- terminal domain that extends up to 200–500 nm from the cell surface [11,12]. Normally, MUC1 is expressed on the apical surface of glandular or luminal epithelial cells of almost all tissues including the mammary gland, stomach, lungs, esophagus, duodenum, pancreas, uterus, prostate, and the hematopoietic cells [13,14]. In healthy tissues, the extended hyperglycosylated branches of MUC1 create a physical barrier and prevent pathogenic access, thus protecting the underlying epithelia [15,16]. The extended sugar branches form a mucinous gel by oligomerization and protect the underlying epithelia from desiccation, pH changes, and invading microbes [17]. During translation, MUC1 is cleaved [18,19], and the extracellular domain with tandem repeats (25–100) is bound to the membrane by noncovalent interaction with the C-terminal domain of MUC1 (MUC1-CD) that consists of a short extracellular domain (ED), the transmembrane domain (TM) and the cytoplasmic domain (MUC1-CT). The MUC1 gene encodes a single polypeptide chain which is cleaved by auto-proteolysis process at a sea -urchin sperm protein enterokinase and agrin (SEA) domain to generate two peptide fragments and heterodimeric MUC1 [11,20]. The β subunit or MUC1-C contains a C-terminal cytoplasmic domain (MUC1-CT) with 69 amino acids, a hydrophobic transmembrane domain (TMD) with 28 amino acids and a short extracellular domain (ECD) with 58-amino acids that is noncovalently attached to the N-terminal extracellular domain (MUC1-N) or α subunit [21]. The cytoplasmic tail of MUC1 (MUC1-CT) aids in signal transduction [17,22].

Among different types of glycosylation, O- and N-glycosylations dominate in MUC1 [23]. The MUC1-N subunit in normal cells, consists of a heavily O-glycosylated- VNTR (variable number of tandem repeat) sequence of 20–21 amino acids (PDTRPAPGSTAPPAHGVTSA), which masks the peptide core and protects it from cleavage by proteolytic enzymes, and also prevents it from undergoing clathrin-mediated endocytosis [24]. The molecular weight of MUC1 can vary between 250–500 kDa based on the percentage of glycosylation (in the range of 50–90% of its molecular mass) and the number of tandem repeats [25]. N-glycosylation of MUC1 occurs at five potential sites, one in the ECD of MUC1-CD, and four in the degenerate repeat of MUC1-N [8]. N-glycosylation patterns are important for MUC1 folding, sorting, apical expression and secretion, whereas O-glycosylation is crucial for its biological properties [26,27].

MUC1 glycosylation depends on the tissue of origin and is regulated by a large number of glycosyltransferases. O-glycosylation is initiated by adding N-acetyl-galactosamine (GalNAc) to the VNTR region highly rich in threonine (Thr) and serine (Ser) residues. Following that, a large family of up to 20 distinct polypeptide GalNAc transferases (ppGalNAc-Ts) form the initial O-linked GalNAcα-Ser/Thr structure (Tn antigen) in the endoplasmic reticulum (ER) and ER-Golgi compartments. This forms the initial O-linked GalNAcα-Ser/Thr structure (Tn antigen) [28]. Following the formation of Tn antigen, GalNAc residue can be further modified by various distinct glycosyltransferases and construct different glycan structures of core 1 also known as T or TF (Thompson-Friedenreich) antigen (by addition of Gal residue) and core 3 (by adding GlcNAcβ1-3GalNAcα) and Sialyl-Tn antigen (STn, by addition of sialic acid residue). Glycosylation continues by extension and chain termination by the addition of carbohydrates such as sialic acid [28,29,30].

However, in cancer cells, MUC1 mostly displays hypoglycosylation of the core glycans, like sialyation of Tn and T antigens via sialyltransferase enzymes that lead to premature chain termination [30,31,32,33,34]. MUC1 expression has been shown to be up to 10 times higher in many human carcinomas than in normal tissues, which provides resistance to chemotherapy [34,35,36]. Therefore, antibodies against tumor associated MUC1 are more likely to bind to the antigen on the surface of tumor cells and not MUC1 on the surface of normal cells. This makes tMUC1 a top molecular target to both detect cancers as well as design antibodies against the altered glycopeptide epitopes in the TR domain. These antibodies are also used to design human T cells to target tMUC1, called Chimeric Antigen Receptor T-cells (CAR T cells) [37,38,39].

### 2.2. Role in GI Tumors

MUC1 is overexpressed and aberrantly glycosylated in most human epithelial cancers [40]. The aberrantly glycosylated MUC1 expressed on malignant cells, called the tumor associated MUC1 or tMUC1 renders usually inaccessible MUC1 epitopes open to detection. MUC1 has been a molecule of interest for immunotherapy for a long time. It is a highly overexpressed cell surface antigen and has altered glycosylation in tumors [41]. However, MUC1 has been shown to play a paradoxical role following infections, acting as an anti-inflammatory molecule in healthy cells and as a pro-inflammatory molecule in cancer cells [42]. In 2009, the National Cancer Institute (NCI) had ranked tMUC1 as the second most targetable antigen out of 75 for developing cancer vaccines [43].

MUC1 has been reported to play a role in tumorigenesis by inhibition of cell death and promotion of metastasis [44,45,46]. MUC1 induces signaling through its cytoplasmic domain (MUC1-CT) and binds to the EGFR family of growth factor tyrosine kinases and enhances signaling through ERK activation and cell proliferation [47]. MUC1-CT interacts with β-catenin, stabilizes it and co-activates Wnt signaling [48]. MUC1 overexpression and its interactions with p53 and FO × O3a transcription factor dampen drug-induced apoptosis and resist oxidative cell damage [49,50]. MUC1 also reduces pro-apoptotic signaling via the heat shock protein (HSP) 90, PI3K/Akt and Caspase-8 pathways [45,51,52]. An increase in depolarized MUC1 leads to the disruption of the normal cell-cell and cell-matrix adhesion and increase in cell-endothelial adhesion, allowing increased metastasis in preclinical models [53]. The hypoglycosylated tMUC1 has increased interaction with cell adhesion molecules ICAM-1 and E-selectin, both of which can improve cellular migration and vascular invasion [54]. MUC1 confers drug resistance in pancreatic ductal adenocarcinoma cells by upregulating multidrug resistance genes [55]. MUC1 has also been reported to increase metastasis through the induction of platelet-derived growth factor (PDGF-A) expression by hypoxia inducible factor (HIF)1-α [56] and leads to epithelial-to-mesenchymal transition in pancreatic cancer [57,58]. MUC1 has also been shown to regulate function of transforming growth factor-β (TGF-β) and switch it from a tumor suppressor to a tumor promoter in PDA cells [59,60]. MUC1 is a prognostic factor that marks poor outcome in gastric cancer patients [61]. Expression of MUC1 has also been reported to be significantly correlated to metastasis in colorectal cancer [44].

**Figure 1:**
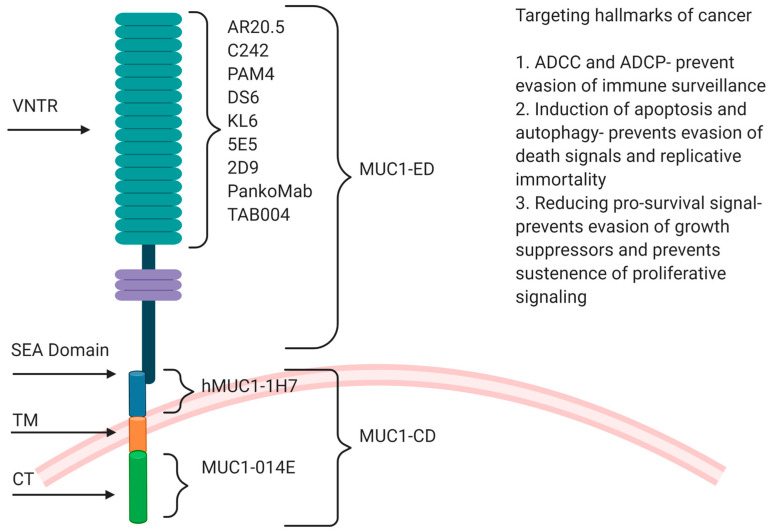
A schematic diagram showing the different antibodies recognizing different domains of MUC1 and also the hallmarks of cancer that they target. The various domains of MUC1 are denoted with different colors, ED and VNTR in sea green, SEA domain in blue, transmembrane domain in orange, and CT in light green.

Overexpression in multiple epithelial tumors, expression all over the surface of a tumor cell due to loss of apicobasal polarity in cancer cells, thus making it accessible to antibodies and tumor-specific aberrant glycosylation with truncated carbohydrate antigens Tn and TF in the VNTR region are features that make MUC1 an attractive target for immunotherapy [37]. Various preclinical and clinical trials have been performed in GI cancers with antibodies against different MUC1 domains (MUC1-N, SEA and MUC1-C), some of them targeting specific hallmarks of cancer (Figure 1).

The objective of this review is to highlight the recent advances made in the treatment of gastrointestinal cancers utilizing antibodies, immunoconjugates and antibody-derived molecular therapies against tMUC1. We have also provided perspectives on how different anti-MUC1 antibodies target different hallmarks of cancer and thus can be utilized as a combination therapy to have better clinical outcomes.

## 3. Anti-MUC1 Antibodies in Preclinical and Clinical Trials

Antibody-based immunotherapy has been used for cancer treatment for the past two decades and is one of the most effective ways to treat hematological malignancies and solid tumors [62,63]. Monoclonal antibodies (mAbs) can be generated by immunizing immunocompetent mice with tumor antigens or tumor cell lysates, or synthetically engineered to bind to specific proteins on cancer cells [64,65]. The fundamental mechanism of therapeutic mAbs are to tag cancer cells for phagocytosis by macrophages or killing by NK or effector T-cells, block the downstream signaling of the target molecule, induce programmed cell death (or autophagy) in the antigen expressing cancer cell, and aid in targeted delivery of therapeutic agents to specifically destroy cancer cells [64,65,66].

Many anti-MUC1 antibodies are in clinical trials or under pre-clinical or experimental studies. The anti-MUC1 antibody-based therapeutics developed against GI cancers that are in pre-clinical and clinical trials have been summarized in Table 1 and Table 2 respectively.

### 3.1. Monoclonal Antibodies

#### 3.1.1. Antibodies Recognizing Non-Glycopeptide Epitope

Human milk fat globule 1 (HMFG1) is an IgG1 murine antibody with kappa light chain, recognizing PDTR epitope within the VNTR region of MUC1-ED. The humanized HMFG1 (AS1402, huHMFG1, Therex, BTH-1704, R-1550) was generated by transferring the complementarity determining regions (CDRs) of the murine HMFG1 onto selected human framework with the same affinity to MUC1 [101,102]. To directly target MUC1 positive advanced pancreatic tumors and trigger neutrophil-mediated immune response, the binding capacity of this mAb in combination with a polysaccharide beta 1,3/1,6 glucan (derived from *S.cerevisiae*) as an immune stimulator with two drugs gemcitabine and Imprime PGG was evaluated [103]. The secondary objectives were to characterize the adverse effects, time to progression, clinical response, progression-free and overall survival. However, this phase Ib trial (NCT02132403) was terminated due to drug recall.

PAM4 is another lgG1 murine mAb, generated by immunizing mice with mucin purified from the xenografted RIP I human pancreatic carcinoma [91]. This mAb can recognize 85% of the pancreatic carcinomas and 50% of the colon carcinomas. However, it does not detect breast, ovarian, renal, prostate and liver cancers [90]. It has been reported that PAM4 is not related to the core epitopes of VNTR and that it also binds to other mucin proteins like MUC5AC [91,104]. In the preclinical studies, 131I- and 90Y-labeled PAM4, was shown to control pancreatic cancer with enhanced survival and clinical responses in pancreatic cancer patients [89,90]. In the phase I clinical trial, 131I-PAM4 IgG and 99mTc-PAM4 Fab′ showed the specific tumor localization in four out of five patients, therefore ensuring these are ideal candidates for further trials [90,105]. Humanized PAM4 (hPAM4, IMMU-107) also known as clivatuzumab was constructed and radiolabeled with Yttrium (90Y) and used for patients with stage III and IV of pancreatic cancer. In a phase I trial, it was shown that 90Y-Clivatuzumab tetraxetan was well tolerated with toxicity restricted to the bone marrow and manageable hematologic toxicity was seen at the maximal tolerated dose of 90Y. Tumor targeting was observed in most patients by using 111In-labeled antibody, and even with mucin antigen present in the serum, there were apparently no issues with the biodistribution or clearance of the antibody. All patients demonstrated disease progression at or after week eight, and some of them had stable target lesions at four weeks after treatment [92]. Hence, combination of chemotherapy and radioimmunotherapy agents was considered for future trials.

Phase I/II trials with 80 participants are ongoing (NCT00603863) to test whether different doses of 90Y-hPAM4 in combination with gemcitabine are safe to give in patients with previously untreated pancreatic cancer. Clinical efficacy of Y-clivatuzumab tetraxetan (DOTA) with or without low-dose gemcitabine (PANCRIT^®^-1) was assessed in a phase I/II/III trial with metastatic pancreatic cancer patients which appeared to be an active first-line therapy for pancreatic cancer [93], but eventually, it was discontinued due to insufficient improvement in overall survival in comparison to placebo [NCT01956812].

GP1.4 is an anti-MUC1 antibody that caused internalization of EGFR in pancreatic cancer cells. This inhibited ERK phosphorylation by EGF stimulation in a MUC1 dependent manner. Inhibition of ERK phosphorylation by GP1.4 resulted in the suppression of proliferation and migration of pancreatic cancer cells [106].

TAB004 is a murine IgG1 mAb that was initially developed by immunizing Balb/c mice with lysates from MUC1-expressing tumors that developed in a human tMUC1 bearing transgenic mouse [77]. TAB004 targets the epitope area with sequence STAPPVHNV present within the TR sequence (AA950-958) of hypoglycosylated tMUC1 [13,17,78,107]. TAB004 distinguishes between normal and tumor-associated forms of MUC1 solely based on the expression of hypo-glycosylated or aberrantly glycosylated MUC1. TAB004 alone or in conjugation with dye-doped mesoporous silica nanoparticles was used to detect breast cancer in vivo [80,108]. TAB004 was also shown to be a diagnostic marker for cancer stem cells and circulating MUC1 in mice and patients with pancreatic cancer [109]. TAB004 in combination with IL2 was shown to improve survival in PDA models by the following mechanisms: (1) reduction in tumor-induced immune regulation and (2) increasing recruitment of CD45+CD11b+ cells, thus increasing antibody-dependent-cellular-cytotoxicity or antibody-dependent-cellular-phagocytosis (ADCC/ADCP) [79]. It has also been reported that, the TAB004 antibody induces complement-independent growth inhibitory effect on PDA cells and significantly increases the anti-tumor efficacy of chemotherapy drugs like 5-FU, Gemcitabine and Paclitaxel [81]. In another study, humanized TAB004 was conjugated to ^111^In and ^225^Ac-DOTA and this immunoconjugate not only could target the tumor specifically but also showed complete preclinical response in triple negative breast cancer [110].

MUC1-014E is another anti-MUC1 antibody raised against an intracellular nonrepeating 19-amino-acid sequence (RYVPPSSTDRSPYEKVSAG) of the MUC1-CT, using a synthetic peptide with the 7-amino-acid epitope (STDRSPY). MUC1-014E showed sharp and specific staining of carcinoma cells, but no staining in fibroblasts, endothelial cells, and inflammatory cells. High rates of positive immunohistochemical staining (97–100%) was found in 107 gastrectomy specimens compared with the other MUC1-related antibodies (MUC1-DF3, MUC1-Ab-5 and PAb anti-MUC1*1110-ecd). MUC1-014E also recognized isolated cancer cells of signet-ring cell carcinoma (sig) and non-solid type poorly differentiated stomach adenocarcinoma (por2). Therefore, this mAb could be used to detect cells in scirrhous gastric cancer [111].

hMUC1-1H7 is an anti-hMUC1 murine mAb developed against a recombinant MUC1 obtained from the breast cancer cell MCF7. It significantly reduced proliferation of breast cancer cells in which it is internalized and specifically localized in MUC1-expressing tumors in the xenograft mouse models. hMUC1-1H7 is specific for the extracellular domain of MUC1-CD and can bind to shed MUC1 as well [76]. It has also been reported that, G3 can inhibit EGF-mediated ERK phosphorylation and cyclin D1 expression, thus, inhibiting EGFR signaling pathways in pancreatic cancer models [75].

#### 3.1.2. Antibodies Recognizing Glycopeptide Epitopes

PankoMab is a murine IgG1, kappa light chain mAb recognizing tMUC1 glycopeptide. It has shown a reduced rate of binding to circulating tMUC1 and mononucleated cells in the serum of colon and pancreatic cancer patients [94]. There are various chimeric and humanized formats of PankoMab under clinical trials as suitable candidates for therapeutic and diagnostic applications [95]. PankoMab-GEX™ (PMG) also known as Gatipotuzumab (previously known as PankoMab-GEX™), is a glyco-optimised mAb with many advantages. For example, it has higher tumor specificity and affinity with an increased number of binding sites, reduced binding to shed MUC1 from colon and pancreatic carcinoma, no binding to peripheral blood mononucleated cells, stronger ADCC, and rapid internalization compared to other antibodies [95]. Its mechanisms of action include ADCC and ADCP. A phase I study in patients with tMUC1 positive advanced solid tumor showed that PMG was safe, well tolerated and showed promising anti-tumor activity [96]. The phase 2 study evaluated the efficacy and safety of PMG’s maintenance therapy compared to placebo in patients with recurrent ovarian, fallopian tube or primary serous peritoneal cancer [97]. This randomized double blinded study reported that PMG failed to improve the time without disease recurrence when given as a single entity [97]. However, it showed a good safety profile, hence, targeting tMUC1 by this antibody in combination with other standard chemotherapy or developing a bi-specific antibody to modulate the immune system holds promise to improve its anti-tumor efficacy [97].

AR20.5 (BrevaRex) is a murine monoclonal antibody (IgG1) developed by immunizing mice with three different sources including MUC1 derived from an ovarian cancer patient, human fluids and MCF-7 cell culture medium. It reacts with six amino acids within the VNTR region (DTRPAP). However, addition of a single GalNAc enhanced the binding affinity of AR20.5 to the MUC1 epitope [24]. AR20.5 forms a complex with circulating MUC1 and/or transmembrane MUC1 on tumor cells. This complex can be internalized by dendritic cells which facilitates effective antigen-processing and cross-presentation of MUC1 to T cells, and leads to the activation of cytotoxic T cells to kill the tumor [112]. In the phase I trial of AR20.5 patients with advanced adenocarcinoma were treated, it induced MUC1-specific immune responses, did not have dose-limiting toxicity, and induced no hypersensitivity reactions. The 2-mg dose showed the strongest biological activity, and was evaluated in future trials [100]. The combination of AR20.5, anti-PD-L1 antibody and PolyICLC rejected human MUC1 expressing tumors and provided a long-lasting, MUC1-specific cellular immune response, which when adoptively transferred to human MUC1 transgenic (MUC.Tg) mice, provided protection against tumor formation. CD8+ cells were found to be the effectors for the MUC1-specific immune response generated by this combination. In the US, a phase I/II clinical trial is ongoing for pancreatic cancer by OncoVent Co., Ltd., with this combination [99].

The DS6 antibody is an IgG1 murine antibody recognizing the CA6 sialoglycotope of tMUC1 that is overexpressed in a variety of solid tumors, including ovarian, breast, cervical, pancreatic and lung cancers. DS6 detects a CA6 antigen that is different from well-characterized tumor-associated antigens, such as MUC1, CA125 and the histo-blood group–related antigens sLea, sLex and sTn [113]. DS6 specifically binds to the tandem repeat domain of CA6-positive MUC1 based on the presence of mucin type O-linked glycans with α2,3-sialylated and β1,4-galactosylated termini [114]. Humanized DS6 (huDS6) antibody was conjugated to the cytotoxic maytansinoid derivative drug DM4 through a cleavable linker. The ADC was called SAR566658 and it showed antitumor efficacy against CA6-positive human pancreas, cervix, bladder, and ovary in vivo tumor xenograft models, with a minimal effective dose correlating with CA6 expression as well as better efficacy than standard-of-care nontargeted tubulin binders. SAR566658 was used in a phase I clinical trial with 114 patients with refractory solid tumors. It showed a satisfactory safety profile and antitumor activity. Tumor improvement was shown in 35–60% of patients at different dosages of SAR566658 [115].

The monoclonal IgG1-kappa antibody C242 was developed by immunizing a mouse with human colorectal adenocarcinoma cell line COLO205. Humanized C242 (HuC242 or Cantuzumab) has the CA242 epitope and reacts with a novel glycoform of MUC1 also known as CanAg glycoprotein (cancer antigen) [83]. CanAg is very highly glycosylated, rich in fucose and sialic acid and Hx-CanAg (heavy subunit) is very similar to MUC1 in amino acid composition, but L-CanAg (light subunit) is different. Deglycosylated H-CanAg can be recognized by the monoclonal antibodies SM-3 and HMFG-2 [84]. Also, due to its high expression in most pancreatic, biliary and colorectal cancers, CanAg is a potential candidate for mAb-based therapies. In a phase I trial, Cantuzumab was conjugated to an anti-microtubule agent mertansine (DM1) and different doses were used to treat colon and rectum carcinomas or other malignancies with positive CanAg antigen as a single intravenous infusion. Results showed that HuC242-DM1 is safe and well tolerated with effective antitumor activity [85,86]. In another phase I trial, cantuzumab conjugated to potent cytotoxic maytansinoid drug ravtansine (DM4), called IMGN242, was found to be well tolerated in colorectal and pancreatic cancer patients at 168 mg/m^2^ dose. This provided a basis to perform phase II clinical studies [87]. The phase II trial was started in CanAg-expressing gastric cancer patients at a dose of 168 mg/m^2^. The data has been amended to differentiate the administered dose of IMGN242 based on the patient’s plasma CanAg levels [88].

KL-6 is a mouse IgG1 mAb that specifically recognizes a sialylated sugar of Krebs von den Lugen-6 (KL-6), which is considered a MUC1-derived glycoprotein antigen. The minimal antigenic epitope for binding of this antibody is PDTRPAP. It has been reported that anti-KL-6/MUC1 mAb increased aggregation of MUC1 glycoproteins at one pole of the cell, called capping of MUC1 on the surface and facilitated E-cadherin-mediated cell-cell interaction in breast cancer cell lines YMB-S and ZR-75-1S. Anti-KL-6 also enhanced the cytotoxic activity of lymphokine-activated killer (LAK) cells. The mechanism of action of this antibody is capping of MUC1 and restoring cell–cell adhesion by E-cadherin, which induces cell cycle arrest by upregulation of the cyclin-dependent kinase, p27 [116]. This also leads to increased accessibility for effector cells to kill tumor cells [67,68,69]. 99mTc labeled anti-KL-6/MUC1 antibody was shown to be a tumor-specific radiotracer that detects pancreatic cancer in vivo, but no further information is available [117].

MY.1E12 is another murine anti-human MUC1 mAb that binds to MUC1 bearing sialylated O-linked oligosaccharides. MY.1E12 was generated by immunizing mice with HMFG. It can identify colon carcinoma tissue [70,71]. MY.1E12 specifically reacts to T structure (ST) attached to Thr8. The sialylation of the T structure (ST) enhances its reactivity with MUC1 [72]. ICG-N-hydroxysulfosuccinimide ester (ICG-sulfo-OSu) and 3-ICG-acyl-1,3-thiazolidine-2-thione (ICG-ATT) were developed as infrared fluorescent-labeling reagents, and anti-human CEA antibody and FMY.1E12 were labelled with 3-ICG-acyl-1,3-thiazolidine-2-thione. This was shown to recognize the gastric cancer tissue specimens with a strong fluorescent signal [73].

5E5 and 2D9 are mouse IgG1k mAbs that were generated by immunization of wild-type Balb/c mice with GalNAc-glycosylated MUC1 glycopeptide (VTSAPDTRPAPGSTAPPAHG) conjugated to KLH. These antibodies exhibited high selectivity for MUC1 tandem repeat glycopeptides with Tn and STn O-glycans and showed preference for Tn-MUC1 glycoforms that had the highest O-glycan occupancy. They can bind to MUC1 with Tn or STn in the GSTA sequence of tandem repeats but do not bind to the GSTA epitope carrying T [74].

### 3.2. Bispecific Antibodies for MUC1

Bispecific antibodies (bsAbs) can recognize two distinct epitopes or antigens simultaneously and therefore enhance the ability of immune cells to engage to tumor cells. Recently, MUC1 has been considered for designing bsAbs.

MUC1-CD16-Bi antibody is a novel bispecific antibody generated via a Serine-Glycine linkage between single domain antibodies (VHH segments) against tMUC1, and CD16 presented on natural killer (NK) cells. The bsAb against MUC1 named MUC1-Bi-1 was humanized by grafting the CDRs of both segments to DP-47 V-segment. Both MUC1-Bi-1 and its humanized version specifically detected tMUC1 on several cancer cell lines (SKOV3, HT29, and LS174) and potentially introduced them to NK cells. These bsAbs had no binding affinity and cytotoxicity to MUC1 negative CHO and HepG2 cells even in the presence of NK cells [118,119].

Different types of bsAbs were constructed with binding affinity to both tMUC1 and CD3 on T-cells. Fab’-S-NB fragments of OKT-3 mAb (anti-CD3) and Fab-SH fragments of MUSE11 mAb (anti-tMUC1) were used to generate the first bsAb which increased the antitumor activity of CD3+ T-LAK cells. MUSE11 is a mouse IgG1 mAb developed against the ascites fluid of gastric cancer patients. The epitope of this antibody could be within the amino acid sequence PDTRPAPG of tMUC1 [120]. MUC1 × CD3 BsAb was constructed with MUSE11 (anti tMUC1) and OKT-3 (anti-CD3), and MUC1 × CD2S BsAb was constructed with MUSE11 and 15E8 (anti-CD28) antibodies. The Fab’-SH from MUSE11 and Fab-S-NB of mouse IgG1 15E8 (anti-CD28) antibodies were used. These BsAbs showed growth inhibition of TFK-1 cancer cells and bile duct carcinoma in SCID mice [121]. The BsAbs (MUC1 × CD3 BsAb and MUC1 × CD28 BsAb) together exhibited 60% cytotoxicity in vitro, similar to that shown by BsAb (MUC1 × CD3) alone. Although reduction in tumor growth was limited, simultaneous administration of a combination of three bsAbs (M × 3, M × 28 and M × 2 bsAb) with peripheral blood mononuclear cells (PBMCs) or T-LAK cells in vitro showed higher cytotoxicity against MUC1-expressing bile duct carcinoma cells [121].

Mx3 diabody is a recombinant BsAb generated using the variable domains of two mAbs directed at effector cells, one against CD3 (OKT-3, mouse IgG2a) and the other against CD28 (l5E8, mouse IgGl), and MUSE11 (mouse IgGl), directed at tMUC1 [122]. One chain consists of a variable heavy chain specific for MUC1 linked to a variable light chain specific for CD3 with a short polypeptide linker GlyGlyGlyGlySer (GGGGS). The second chain has a variable light chain specific for MUC1 linked to a variable heavy chain specific for CD3. Therefore, Mx3 diabody can specifically bind to both MUC1 and CD3 positive LAK cells with a T cell phenotype (T-LAK). Mx3 diabody with T-LAK showed growth inhibition in about 98% of TFK-1 cells with an effector:target ratio of 10 [122]. Mx3 was fused genetically to the mutated superantigen staphylococcal enterotoxin A (SEA) D227A to specifically target bile duct carcinoma (BDC). This super-antigen fused diabody also showed the potential to inhibit the BDC cell line TFK-1 and reduce tumor size when compared to the Mx3 diabody alone [123].

A bsAb containing F(ab′)2/F(ab′) fragments with a functional chemical linker is the anti-MUC1/anti-Ga chelate. A mouse IgG1 12H12 mAb raised against a mouse glycosylated form of MUC1 called TAG-12 was combined to another mouse IgG3 anti-Ga chelate mAb. Prior to 3A10 F(ab′) coupling, the 12H12 F(ab′)2 fragment was labeled with 125I. This bispecific-mAb showed improved immunoscintigraphic tumor localization in breastcarcinoma bearing mice [124].

Another bsAb has been constructed with a novel PD-1 inhibitor-induced cytokine- induced killer cells (CIKs) armed with an anti-tMUC1 and anti- CD3 antibodies. This bsAb is currently under several phase II randomized clinical trials for advanced gastric, kidney, lung, breast, colorectal, pancreatic and liver cancers, but there is no further information available ([NCT03554395], [NCT03540199], [NCT03501056], [NCT03524261], [NCT03524274], [NCT03509298], and [NCT03484962]) [98].

### 3.3. CAR-T Cells Targeting MUC1

TAB004 has been used to make a CAR-T cell construct, which has exhibited significant cytotoxic activity against pancreatic cancer cells and reduced growth of orthotopic pancreatic tumors in a NOD-SCID mouse model [82]. Some PDA cells, for example CFPAC and HPAF II, were found to be resistant to the therapy and several genes were overexpressed in them such as indoleamine 2, 3-dioxygenases-1 (IDO1), cyclooxygenase 1 and 2 (CO × 1/2), and galectin-9 (Gal-9) [82]. This study showed that combining biological inhibitors of IDO1, CO × 1/2, and Gal-9 with the CAR-T cells resulted in significant enhancement of CAR-T cell cytotoxicity against PDA cells.

5E5 mAb showed high specificity to breast cancer cells and tissue [37,123] and was used to develop MUC1 CAR-T cells. These CAR-T cells showed cytotoxicity against leukemia and pancreatic cancer cells and also enhanced survival of mice by eliminating the barriers for engagement of the endogenous immune system [38,125].

## 4. Molecular Interactions between MUC1 and Its Antibodies

X-ray crystallography of antibody crystal structures [126] and NMR analysis of glycopeptides [127] are used to understand the biochemical interactions or molecular recognition between the antigen and antibody. The Tn antigen is one of the most important structural motifs of tMUC1 found widely in many different aggressive carcinomas [128,129]. It has been shown by years of extensive effort to develop antibodies targeting tMUC1 having the Tn antigen, that most anti-MUC1 antibodies do not directly bind to carbohydrates. However, the binding affinities with the immunodominant MUC1 are shown to be significantly increased by O-glycosylation in this area [130,131,132]. AR20.5 bound to the glycopeptide with stronger affinity than the naked peptide. These observations led to the hypothesis that the antibody must specifically bind the carbohydrate as well as the peptide. X-ray crystallography of the structures of AR20.5 [24] and SM3 [133] in complex with both peptide and glycopeptide revealed that the carbohydrate did not have any specific polar contacts with the antibody. The high affinity for the glycopeptide and the lack of specific binding contacts of AR20.5 suggest that glycosylation of MUC1 stabilizes an extended bioactive conformation of the peptide that is recognized by the antibody. Evidence suggests that glycosylation of the peptide alters the conformational equilibrium of the antigen, and this allows the antibody to select the correct conformation. Therefore, glycosylation of MUC1 is important for the generation of high affinity therapeutic antibodies [24]. The anti-MUC1 KL-6 antibody distinguishes between the ST, Tn, and T antigens at the same O-glycosylation site independent of the modifications at other potential sites [68,131,132]. The NMR study suggests that KL6 mAb strictly recognizes the epitope from the extended trans conformation of a glycopeptide, which has been modified with the ST antigen. Detailed molecular recognition studies on MUC1 and anti-MUC1 antibodies and the use of synthetic glycopeptide library to develop a new class of antibodies targeting “dynamic glycopeptidic neoepitopes” with disease-relevant O-glycosylation in immunodominant mucin domains have been described recently [134].

The lack of carbohydrate-binding specificities in most anti-MUC1 mAbs is a huge challenge for the development of MUC1-based therapeutic antibodies. Antibodies binding to cancer-relevant glycopeptidic neoepitopes with higher specificities in carbohydrate recognition will be beneficial in the development of anti-MUC1 mAbs as therapeutic and diagnostic agents in the clinical settings.

## 5. Concluding Remarks and Future Perspectives

In spite of MUC1 being a top target, multiple trials with MUC1 antibodies and antibody-derived immunotherapies have failed to translate to the clinic. Most of the trials have been discontinued for not being sufficiently effective. There may be various reasons for the inefficiency of the antibodies. As of now, many anti-MUC1 antibodies have been developed against the highly immunogenic VNTR region of MUC1 α chain (MUC1-ED) [135]. After cleavage at the SEA domain, the MUC1-N is often shed from the surface of cells and released into the peripheral blood. The shed α subunits (MUC1-N) sequester anti-MUC1 antibodies against the VNTR region, preventing them from binding to the surface MUC1 [95]. To overcome this problem, antibodies against MUC1-CD could be used as a more effective strategy. Shedding of MUC1-N increases its levels in the serum of patients with various cancers [136,137,138], thus, reducing the specificity and effective binding of the antibodies to MUC1 on the tumor cells [136,139]. Therefore, serum levels of MUC1 in different cancer patients need to be evaluated to find an effective dose of the antibodies [140]. In addition, bsAbs can be made by combining immune checkpoint inhibitors such as anti-PD1 and anti-PD-L1 antibodies with anti-MUC1 antibodies. This will increase engagement of the immune cells with the tumor. In recent years, antibodies are being designed against the other domains of MUC1 including SEA, extracellular, and intracellular MUC1-CT. Therefore, rational designing of antibodies and combination therapy strategies are important to achieve a good safety and efficacy profile against MUC1 expressing cancers.

Antibodies to the non-glycopeptide part of the VNTR region have not been able to generate an effective cellular or humoral immune response to tMUC1 [141]. Antibodies to MUC1 peptide also do not effectively recognize MUC1-expressing tumor cells. However, antibodies raised against shortened glycopeptide structures with a simple T antigen (T, Galβ1-3GalNAc), sialyl Tn (NeuAcα2-6GalNAc) and Tn (GalNAc) elicit the strongest immune response against MUC1-expressing tumor cells [142]. This happens due to the specific presence of Tn and STn glycans on MUC1 expressing cancer cells, but not on normal epithelial cells and the blocked regions of the VNTR domain get exposed to recognition by antibodies, thereby, producing tumor-specific recognition sites. As evident, studying the glycosylation changes have led to the development of potentially effective MUC1-based immunotherapy [143,144]. Some anti-MUC1 antibodies can recognize the MUC1 epitopes on both normal epithelial and tumor cells thus compromising the specificity [145]. Also, heterogeneity of MUC1 expression levels, the glycosylation pattern and subcellular distribution contribute to reduced binding efficiency. The different glycoforms may confer an evolutionary advantage on the tumor cells to be resistant against antibody-based therapies [145,146]. Therefore, a combination of antibodies that can detect many glycoforms of MUC1 can be considered for clinical trials.

Anti-MUC1 antibodies directed against the SEA domain target the junction of MUC1 α and β subunits, which is composed of intact epitopes from both [147,148]. These anti-SEA domain antibodies have shown high affinity and effectivity compared to antibodies targeting the VNTR region [148].

The mechanism of action anti-MUC1 mAbs target one or more hallmarks of cancer. For example, some antibodies have been reported to show ADCC and ADCP, some others block anti-apoptotic mechanisms thus inducing cell death, also some antibodies reduce expression of pro-survival genes. Gatipotuzumab is a glycooptimized antibody developed by Glycotope’s GlycoExpress™ platform that significantly improved treatment outcome with mechanisms such ADCC, tumor cell phagocytosis and induction of apoptosis compared to non-glycooptimized biotherapeutics [97]. Other antibodies against MUC1 glycopeptide, such as 5E5 and 1B2, have been shown to be effective as immunotherapy strategies because of their high specificity to tMUC1 and ability to induce ADCC [149]. Therefore, by utilizing the mechanism of action of an antibody, strategies could be developed to eliminate the tumor.

However, a decrease in concentration of anti-MUC1 antibodies targeting the tumor and their poor internalization due to the extracellular MUC1-N barrier remain major hurdles. To overcome this, development of antibody fragments can be considered [143,150]. Also, a whole or fragmented antibody could be conjugated to potent drugs to target specific types of tumor cells. For example, Napabucasin, which is a STAT-3 inhibitor was under Phase III clinical trials for PDA but was discontinued due to futility [151]. However, it has been shown that high-MUC1 PDA cells are more sensitive toward the STAT-3 inhibitor Napabucasin [152]. Therefore, anti-MUC1 antibodies armed with Napabucasin may be a promising strategy to eliminate high-MUC1 tumors. Bispecific and trispecific antibodies armed with anti-PD-1, anti-MUC1 and anti-CD3 are new products under clinical trials [98].

## Figures and Tables

**Table 1:** MUC1 antibodies under preclinical trials for GI cancers.

Antibody	Epitope	Original Antigen	Treatment under Trial	GI Cancer Type	Year	Reference
KL-6	a sialylated sugar of Krebs von den Lugen-6 (KL-6) PDTRPAP sequence	a sialylated sugar of Krebs von den Lugen-6 (KL-6) PDTRPAP sequence	99mTc labeled anti-KL-6/MUC1	Pancreatic Cancer	2008	[67,68,69]
MY.1E12	sialyla2–3galactosylh1-3Nacetylgalactosaminide linked to a distinct threonine residue in the MUC1 tandem repeat	HMFG	3-ICG-acyl-1,3-thiazolidine-2-thione labeled MY.1E12	Gastric Cancer	2008	[70,71,72,73]
5E5, 2D9	Tn or STn in the tandem repeat domain	GalNAc-glycosylated MUC1 glycopeptide (VTSAPDTRPAPGSTAPPAHG) conjugated to KLH	5E5 MUC1-CAR-T cells	Pancreatic Cancer	2016 2019	[74]
hMUC1-1H7	extracellular domain of MUC1 C-terminal subunit (MUC1-C)	recombinant human (rh) protein including extracellular region of MUC1- C (rhMUC1-EC192) obtained from MCF7 cells	hMUC1-1H7	Pancreatic Cancer	2004	[75,76]
TAB004	STAPPVHNV within the TR sequence	Protein lysate from MUC1-expressing tumors that developed in a MUC1 transgenic mice (PDA mice) that expressed human MUC1	(1) TAB 004 (2) CAR-T cell therapy (3) Bispecific antibody with anti-CD3	Pancreatic Cancer	2008–2019	[77,78,79,80,81,82]

**Table 2:** MUC1 antibodies under clinical trials for GI cancers.

Antibody	Epitope	Original Antigen	Treatment under Trial	GI Cancer Type	Clinical Trial Status	Year	Reference
huC242	Sialyl-Lewis a epitope CanAg glycoprotein which is similar to MUC1	Human colorectal adenocarcinoma cell line COLO205	huC242-DM4	(1) Non-colorectal Cancer, Pancreatic Cancer (2) Locally Advanced and metastatic Stomach, Gastric and other GI cancers	(1) Phase I completed (2) Phase II withdrawn	2006 2008	[83,84,85,86,87,88]
huPAM4	Domain located between the amino terminus and start of the repeat domain of a MUC1 antigen (non- VNTR) and also react with MUC5AC	Mucin purified from the xenografted RIP I human pancreatic carcinoma	111In-huPAM4	Pancreatic Cancer	Phase I terminated	2006	[89,90,91]
hPAM4 (Clivatuzumab)	Domain located between the amino terminus and start of the repeat domain of a MUC1 antigen (non-VNTR) and also react with MUC5AC	Mucin purified from the xenografted RIP I human pancreatic carcinoma	(1) 90Y-hPAM4 (Clivatuzumab) (2) 90Y-hPAM4-Tetraxetan & Gemcitabine vs. Placebo & Gemcitabine	(1) Pancreatic Cancer (2) Metastatic Pancreatic Cancer	(1) Phases I and II completed (2) Phase III terminated	2008 2013	[92,93]
SAR56665 8huDS6-DM4	O-linked glycans with α2,3-sialylated and β1,4-galactosylated termini in VNTR	Human serous ovarian carcinoma	SAR56665 8huDS6-DM4	Pancreas	Phase II completed	2010	
PankoMab-GEX™ (Gatipotuzumab)	Epitope...PDT*RP..., where T* is O-glycosylated with GalNAca1- or a similar short, non-sialylated glycan such as Galb1-3GalNAca1-(core-1)	Tumor MUC1 from a desialylated human breast cancer source	(1) PankoMab-GEX™ (Gatipotuzumab) (2) Combination of Gatipotuzumab and anti-EGFR Tomuzotuximab	(1) Pancreatic (2) Colorectal	(1) Phase II, ongoing (2) Recruiting for Phase I	2010 2017	[94,95,96,97]
PD-1 inhibitor armed with an anti-MUC 1 and anti- CD3 bispecific antibody	Information unavailable	Information unavailable	PD-1 inhibitor armed with an anti-MUC 1 and anti- CD3 bispecific antibody	Advanced Gastric, Colorectal, Pancreatic and Liver cancers	Recruiting for Phase II	2018	[98]
AR20.5	DTRPAP and DTnRPAP	MUC1 from an ovarian cancer patient, derived from human fluids and breast cancer cell MCF-7 culture medium	(1) AR20.5 (2) Combination of mAb-AR20.5, anti-PD-L1 and Poly ICLC	(1) Advanced adenocarcinoma (2) Pancreatic Cancer	(1) Completed Phase I (2) Phase I/II ongoing	2004 2018	[24,99,100]
